# Probing non-perturbative QED with electron-laser collisions

**DOI:** 10.1038/s41598-019-45582-5

**Published:** 2019-06-28

**Authors:** C. Baumann, E. N. Nerush, A. Pukhov, I. Yu. Kostyukov

**Affiliations:** 1 Institut für Theoretische Physik I, Heinrich-Heine-Universität Düsseldorf, 40225 Düsseldorf, Germany; 2 Institute of Applied Physics, Russian Academy of Sciences, 603950 Nizhny Novgorod, Russia; 3 Lobachevsky State University of Nizhny Novgorod, 603950 Nizhny Novgorod, Russia

**Keywords:** Laboratory astrophysics, Laser-produced plasmas, Computational science, High-field lasers, Nonlinear optics

## Abstract

The vast majority of QED results are obtained in relatively weak fields and so in the framework of perturbation theory. However, forthcoming laser facilities providing extremely high fields can be used to enter not-yet-studied regimes. Here, a scheme is proposed that might be used to reach a supercritical regime of radiation reaction or even the fully non-perturbative regime of quantum electrodynamics. The scheme considers the collision of a 100 GeV-class electron beam with a counterpropagating ultraintense electromagnetic pulse. To reach these supercritical regimes, it is unavoidable to use a pulse with ultrashort duration. Using two-dimensional particle-in-cell simulations, it is therefore shown how one can convert a next-generation optical laser to an ultraintense (*I* ≈ 2.9 × 10^24^ Wcm^−2^) attosecond (duration ≈ 150 as) pulse. It is shown that if the perturbation theory persists in extreme fields, the spectrum of secondary particles can be found semi-analytically. In contrast, a comparison with experimental data may allow differentiating the contribution of high-order radiative corrections if the perturbation theory breaks.

## Introduction

The invention of chirped pulse amplification^1^ almost 30 years ago has led to ever stronger laser systems. State-of-the-art systems are already able to deliver peak intensities of the order of 10^22^ Wcm^−2^ ^2–4^ and future projects aim at reaching even higher intensities^5–7^. Consequently, the perspectives are opened to explore new regimes of laser-matter interactions. Essentially, the interaction of an electromagnetic (EM) field with an electron can be characterized by two Lorentz invariant quantities^8^: (i) the dimensionless vector potential ${a}_{0}=|e|\sqrt{-{A}_{\mu }{A}^{\mu }}/({m}_{e}c)$ and (ii) the quantum parameter $\chi =\sqrt{-{({F}_{\mu \nu }{p}^{\nu })}^{2}}/({m}_{e}c{E}_{{\rm{crit}}})$. Here, *e* and *m*_*e*_ are the electron charge and mass, respectively, *c* is the speed of light, *A*_*μ*_ is the four-vector potential, *F*_*μν*_ is the EM field tensor, *p*^*μ*^ is the four-momentum and *E*_crit_ is the critical field of quantum electrodynamics (QED), *E*_crit_ ≈ 1.3 × 10^16^ Vcm^−1^ ^9,10^. Simply said, *a*_0_ can be seen as a measure for relativistic effects (here *a*_0_ ≫ 1), while *χ* accounts for the impact of quantum effects. Especially the investigation of strong-field quantum effects, like the recoil due to emitted photons (radiation reaction) and the pair production according to the Breit-Wheeler process^11^, is of fundamental interest. These regimes become important when *χ* approaches unity and with nowadays experiments one can already probe the regime $\chi \mathop{ < }\limits_{ \tilde {}}1$ with optical fields^12–15^ or with the field of crystals^16^ which even allows one to reach *χ* ≈ 7^17^. However, although not yet realizable in laboratories, it is still important to study situations in which $\chi  \ggg 1$, referred to as “supercritical regime”^18^ in the following. Such systems are physically relevant, for example for the understanding of extreme astrophysical environments^19–21^. An even more extreme regime is $\alpha {\chi }^{\mathrm{2/3}}\mathop{ > }\limits_{ \tilde {}}1$ (*α* ≈ 1/137 is the fine-structure constant) that has long been assumed to be not accessible experimentally. In this regime, it is believed that quantum processes will be dominated by radiative corrections. This means the emission of virtual photons by an electron or the virtual conversion of a photon in an *e*^−^*e*^+^ pair cannot be treated as a small perturbation and in this sense QED becomes a fully non-perturbative theory^22–24^. So far, it does not exist a closed theory describing this highly nonlinear regime, and there are only a couple of works that have studied this regime over the last decades^24–26^.

This manuscript shows that it might be possible to probe the regime $\alpha {\chi }^{\mathrm{2/3}}\mathop{ > }\limits_{ \tilde {}}1$ in an electron-EM pulse collider setup in the future. Considering an ultrarelativistic electron, the quantum parameter can be written approximately as *χ*_*e*_ ≈ *γ*_*e*_*F*_⊥_/(|*e*|*E*_crit_), where *γ*_*e*_ is the Lorentz factor of the electron and *F*_⊥_ is the force acting perpendicular to its velocity. In terms of the quantum parameter *χ*_*e*_, the fully non-perturbative QED regime sets in at *χ*_*e*_ ∼ 1600. Assuming an electron with *γ*_*e*_ = 2.5 × 10^5^ (≈125 GeV energy), a field strength of 8.3 × 10^13^ Vcm^−1^ is required to reach this completely novel regime of QED. In principle, the next generation of laser systems will deliver such extreme field conditions^5–7^. However, discussing the characteristic time between two photon emission events in the case *χ* ≫ 1^27,28^,

1 $$\begin{array}{l}{t}_{{\rm{rad}}}\sim {W}_{{\rm{rad}}}^{-1}={(1.46\frac{{m}_{e}{c}^{2}}{\hslash {\gamma }_{e}}\alpha {\chi }_{e}^{\mathrm{2/3}})}^{-1}\sim 200\,{\rm{as}},\end{array}$$

one can see that for optical lasers, even a single-cycle pulse, which duration is ∼3 fs, is not sufficiently short (ℏ is Planck’s constant). Electrons will radiate almost their entire energy in terms of high-energetic *γ*-photons before they reach the region of maximal field strength. Consequently, getting access to the regime $\alpha {\chi }_{e}^{\mathrm{2/3}}\mathop{ > }\limits_{ \tilde {}}1$ is not easily achieved with conventional laser pulses of ultra-high intensity. Therefore, it is unavoidable to develop experimental schemes that mitigate these radiative losses significantly. This can be done, for instance, by reducing the interaction time between electrons and the background field. Here, a first approach has been presented by Yakimenko *et al*.^29^. Instead of using strong lasers sources, that work suggests to use a future 100 GeV electron-electron collider. To achieve the required field strengths, the electron beams are supposed to be extremely dense and tightly focused transversely (*σ*_⊥_ = 10 nm), leading to a strong peak current (*I*_max_ = 1.7 MA) and so to strong collective self-fields. Mitigation of radiative losses is ensured by a strong longitudinal compression of the beams (*σ*_∥_ = 10 nm). Another approach how one could probe the impact of radiative corrections has been proposed by Blackburn and coworkers^18^. They raised the idea of colliding highly energetic electrons with ultraintense laser pulses. To mitigate radiative losses of the electrons, they put an oblique scattering geometry forward. In doing so, it seems possible to reach *χ*_*e*_-values of the order of 100. The present manuscript, however, aims at introducing a setup that makes a supercritical regime of radiation reaction and pair production accessible, and that may even be promising for exceeding the *χ*_*e*_-threshold of 1600. In particular, it is discussed how it may become possible to enter the regime $\alpha {\chi }_{e}^{\mathrm{2/3}}\simeq 1$ even in the head-on collision of a high-intensity pulse with a 125 GeV electron beam. Therefore, it is shown how one can convert a next-generation optical laser pulse to an extremely intense attosecond pulse. The observed attosecond duration (∼150 as) is sufficiently reduced to suppress radiative losses significantly. Based on the Ritus–Nikishov theory for strong-field QED processes^30^, numerical as well as analytical calculations are presented to investigate the interaction of ultrarelativistic beam electrons with extremely intense fields. The numerical simulations underline the feasibility of the proposed scheme.

## Results

### Proposed scheme and its numerical modeling

To corroborate the possibility of reaching the fully non-perturbative QED regime in the collision of an electron beam with an ultrashort EM pulse, two-dimensional particle-in-cell (PIC) simulations are performed with the code VLPL^31,32^. The PIC code can be used to incorporate QED events into the self-consistent modeling of the laser-particle interaction. In the simulations, it is therefore possible to account for the emission of *γ*-photons by ultrarelativistic particles via the nonlinear Compton scattering process and for the creation of electron-positron pairs according to the multiphoton Breit-Wheeler process. These processes are included in terms of the widely used Monte-Carlo approach^28,33,34^. A benchmark of the QED module that is implemented into VLPL against a similar code can be found in^35^. Note that the QED module is based on the Ritus–Nikishov formulas which do not account for high-order radiative corrections so far (probably needed at $\alpha {\chi }_{e}^{\mathrm{2/3}}\mathop{ > }\limits_{ \tilde {}}1$). Consequently, it may be that the method models particles in such extreme states only inappropriately. The Ritus–Nikishov results, however, are far apart from being useless. Performing the proposed experiment and comparing the experimental data with the results of the Ritus–Nikishov theory (see the discussion in the following sections) allows important insights in at least two unsolved questions:

(i) Is the Ritus–Nikishov theory applicable in this regime?
(ii) If the Ritus–Nikishov theory is inapplicable in this regime, how will the observables be modified and what can be learned from that?

Thus, the approach presented here is a logical step towards a better understanding of superstrong-field QED.

**Figure 1:**
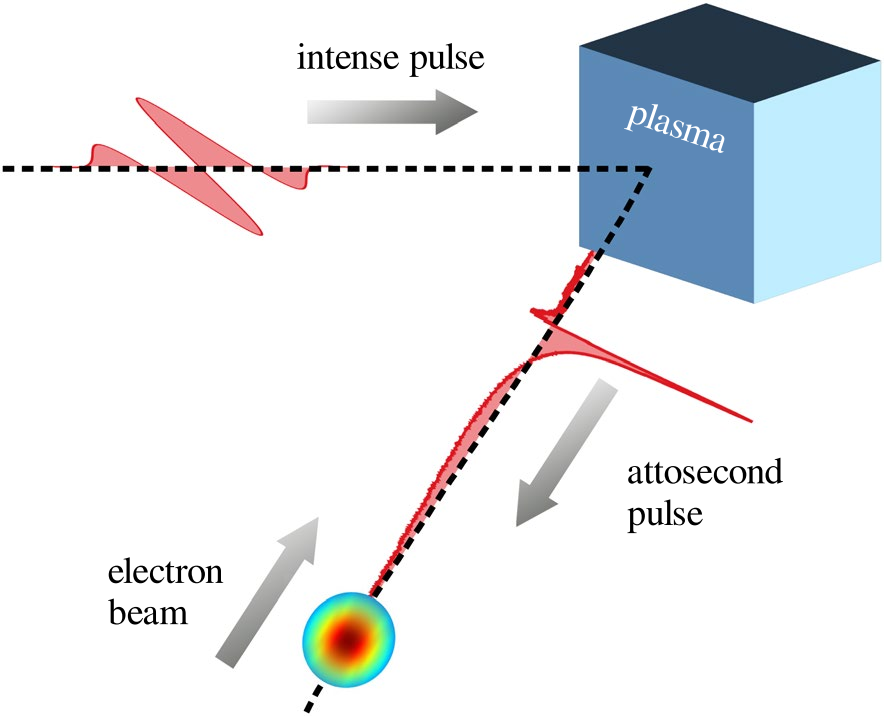
The figure shows a sketch of the proposed scheme. A moderately next-generation laser pulse impinges at oblique incidence (*θ* = 30°) onto a overdense plasma target (150* n*_cr_). The plasma converts the incoming radiation into an ultraintense attosecond pulse via high harmonic generation.

As already pointed out in the introduction, it is necessary to convert the laser radiation into an attosecond pulse with ultra-high intensity. To fulfill this requirement, the proposed scheme follows the approaches previously presented in works considering the generation of high harmonics^36,37^. A p-polarized single-cycle laser pulse (central wavelength *λ*_0_ = 1 *μ*m) impinges at oblique incidence onto an over-dense plasma slab (see sketch in Fig. 1). In principle, a generalization to longer laser pulses is possible when using the attosecond lighthouse technique^38^. The laser has a Gaussian shape in the transverse direction and it is focused to a spot size of *w*_0_ = 2.5*λ*_0_ at the focal point *x*_0_ = 10*λ*_0_, *y*_0_ = 0. The dimensionless vector potential *a*_0_ is set to 350, which corresponds to a peak intensity of 1.68 × 10^23^ Wcm^−2^ and to a total power of 35 PW. The angle of incidence, measured with respect to the target’s normal direction, is equal to *θ* = 30°. The target is modeled by a slab with initial density 150*n*_cr_, where *n*_cr_ = 1.12 × 10^21^ cm^−3^ is the critical density for the above wavelength. It is assumed to be fully ionized initially. The ion mass-to-charge ratio is chosen to be two times that of protons, which means that one could potentially use any fully ionized low-Z species as a target material. It is also important to stress that the plasma slab is attached to a plasma density ramp, modeled as *n* ∝ exp[(*x* − *x*_0_)/(0.33*λ*_0_)] for *x* < *x*_0_. The plasma profile impacts the efficiency of the generation of high harmonics and defines the requirements for the contrast of the high-power laser pulse^39^. The probing electron beam propagates under the same angle *θ* such that it can hit the reflected radiation in a nearly head-on scenario. The beam profile is Gaussian-like with rms length ${\sigma }_{\parallel }={\lambda }_{0}\mathrm{/40}$ and rms width *σ*_⊥_ = *λ*_0_/5. Each beam electron has an initial *γ*_*e*_-factor of 2.5 × 10^5^, corresponding to an energy of roughly 125 GeV. The beam density is determined such that the beam yields a peak current of *I*_max_ ≈ 13.5 kA and a total charge of *Q* ≈ 2.8 pC. Initially, the beam is shifted spatially so that it hits the EM attosecond pulse in its focus. The simulation box has a size of 15*λ*_0_ in the *x* direction and 20*λ*_0_ in the *y* direction with a cell size of Δ*x* = Δ*y* = 0.005*λ*_0_.

### Simulation results

**Figure 2:**
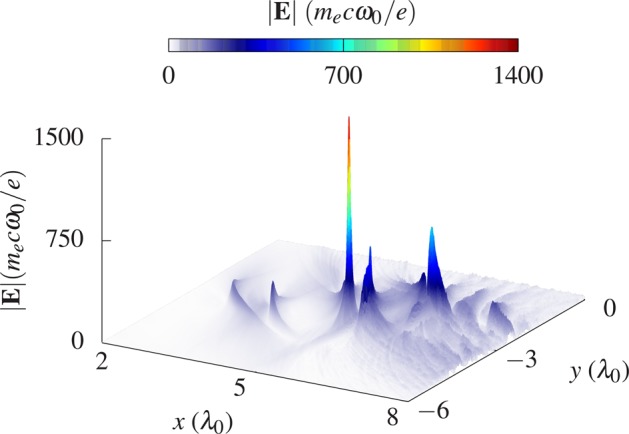
The plot illustrates the generated light pulse in terms of the absolute value of the electric field at time *t* = 13*T*_0_. At this moment in time the pulse reaches its peak value.

**Figure 3:**
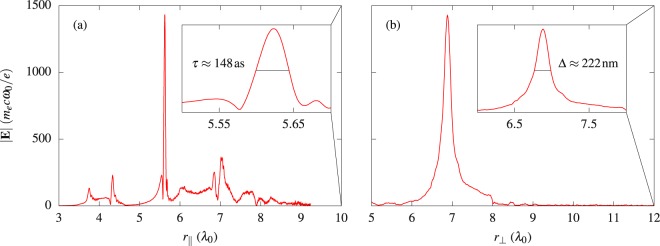
The figure shows a characterization of the (**a**) longitudinal profile of the generated EM pulse, seen by the ultrarelativistic electron beam. The inset shows a zoom of the attosecond pulse, with a FWHM duration of ≈148 as. (**b**) The transverse profile of the attosecond pulse at the focus is shown, indicating a FWHM width of ≈222 nm (inset).

Figure 2 presents the results for the reflected radiation in terms of the absolute value of the electric field at time *t* = 13*T*_0_. At this time instant, the EM field is most tightly focused in the transverse direction and it is most compressed in the longitudinal direction, leading finally to the emergence of an ultraintense and ultrashort EM pulse. A transverse and a longitudinal characterization of the pulse is given in Fig. 3(a and b), respectively. In particular, Fig. 3(a) shows a cut of |**E**| along the propagation axis of the electron beam (angle *θ* = 30°). The length *r*_∥_ stands here for the distance of a point on the propagation axis with respect to the intersection point with the *y*-axis. It can be seen that the main peak of the electric field protrudes from the rest of the field structure and one finds a maximum value of |**E**| ≈ 1450. In addition, the focused pulse appears to be very narrow on the applied length scale. Therefore, the inset shows an enlarged picture of the main peak. One can extract a duration (full width at half maximum, FWHM) of roughly 150 as from the data. Comparing this with the radiation time in Eq. (1) (*t*_rad_ ≈ 200 as), the duration of the attosecond pulse is less than this characteristic time between two photon emission events. Consequently, the attosecond pulse might be sufficiently short to prevent electrons from radiating all their kinetic energy too fast and so making it possible to reach the highest possible values of *χ*. Figure 3(b) displays a cut of the field profile along the direction perpendicular to the beam axis at *r*_∥_ ≈ 5.625*λ*_0_. It can be seen that, besides its ultrashort duration, the generated pulse is characterized by a tight focal width of approximately 220 nm.

**Figure 4:**
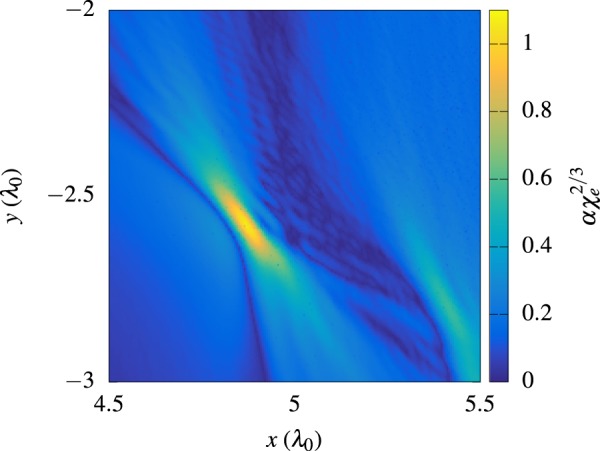
Non-perturbative quantum parameter $\alpha {\chi }_{e}^{\mathrm{2/3}}$ at time *t* = 13*T*_0_. The simulation results indicate that the regime $\alpha {\chi }_{e}^{\mathrm{2/3}}\mathop{ > }\limits_{ \tilde {}}1$ might be accessible in the proposed scheme.

Figure 4 shows the maximum value for the fully non-perturbative QED parameter $\alpha {\chi }_{e}^{\mathrm{2/3}}$ in each simulation cell. It can be seen that the electrons in the vicinity of the focal spot indeed experience such extreme conditions that one could probe the breakdown of perturbation theory. Notably, a maximum value of *χ*_*e*_ ≈ 1750 can be observed in the simulations.

### Model

The following section presents an analytical model for the interaction of ultrarelativistic particles with strong EM fields in the case *χ* ≫ 1. The model describes the evolution of the system in a self-consistent manner since it also takes into account the generation of secondary particles. A comparison with particle spectra obtained from simulations indicates reasonable agreement.

The analytical approach is based on the distribution of particles in the phasespace. The corresponding time evolution of the system is described by the Boltzmann equations (BE, for electrons, photons and positrons; see, for example^28^). The electron trajectories are bent by the EM field only by the negligible angle |*e*|*Eτ*/(*m*_*e*_*cγ*_*e*_) ∼ 10^−3^, thus one can neglect the transverse particle dynamics and proceed to a one-dimensional BE for the particle distribution functions. In this one-dimensional description the external fields are taken at position *x*(*t*) = *x*(0) + *ct*^40^, and the distribution functions are the same as the particle spectra. As will be shown later, the exact dependence of the external field on time does not matter if *χ* ≫ 1, and the global constant field approximation can be applied (see also ref.^17^ for more information). Thus, the BE in a constant homogeneous magnetic field of the strength *H* can be used instead of using an alternating laser field. It yields that the particle’s phasespace evolution is governed by

2 $${\partial }_{t}{f}_{e,p}=-\,{W}_{{\rm{rad}}}{f}_{e,p}+{\int }_{\varepsilon }^{\infty }{f}_{e,p}(\varepsilon ^{\prime} ){w}_{{\rm{rad}}}(\varepsilon ^{\prime} \to \varepsilon )d\varepsilon ^{\prime} +{\int }_{\varepsilon }^{\infty }{f}_{\gamma }(\varepsilon ^{\prime} ){w}_{{\rm{pp}}}(\varepsilon ^{\prime} \to \varepsilon )d\varepsilon ^{\prime} ,$$

3 $${\partial }_{t}{f}_{\gamma }=-\,{W}_{{\rm{pp}}}{f}_{\gamma }+{\int }_{\varepsilon }^{\infty }[{f}_{e}(\varepsilon ^{\prime} )+{f}_{p}(\varepsilon ^{\prime} )]{w}_{{\rm{rad}}}(\varepsilon ^{\prime} \to \varepsilon ^{\prime} -\varepsilon )d\varepsilon ^{\prime} ,$$

where all distribution functions are given at (*t*, *ε*) unless otherwise specified. Additionally, *w*_rad_(*ε*′ → *ε*) is the differential emission probability rate for the electron whose energy drops from *ε*′ to *ε* after the emission of a single photon, *w*_pp_(*ε*′ → *ε*) ≡ *w*_pp_(*ε*′ → *ε*′ − *ε*) is the differential probability rate for a photon with energy *ε*′ to produce an electron with energy *ε* and a positron with energy *ε*′ − *ε*, and ${W}_{{\rm{rad}},{\rm{pp}}}(\varepsilon )={\int }_{0}^{\varepsilon }{w}_{{\rm{rad}},{\rm{pp}}}(\varepsilon \to \varepsilon ^{\prime} )d\varepsilon ^{\prime}$ are the full probability rates for photon radiation and for pair photoproduction, respectively. Since the emission and pair production differential probabilities at $\alpha {\chi }^{\mathrm{2/3}}\mathop{ > }\limits_{ \tilde {}}1$ are not yet known, the Nikishov–Ritus (Baier–Katkov) probabilities^27,28,30^ are used instead in the following.

To further advance the analytical calculations, one first considers energies of the secondary particles that correspond to *χ* ≫ 1. In this regime, the differential probabilities can be written as^41^

4 $${w}_{{\rm{rad}}}(\varepsilon ^{\prime} \to \varepsilon )=\frac{{\rm{\nu }}}{{\varepsilon ^{\prime} }^{\mathrm{4/3}}}\frac{1+{\eta }^{2}}{{\eta }^{\mathrm{1/3}}{\mathrm{(1}-\eta )}^{\mathrm{2/3}}}{(\frac{H}{{H}_{{\rm{crit}}}})}^{\mathrm{2/3}},$$

5 $${w}_{{\rm{pp}}}(\varepsilon ^{\prime} \to \varepsilon )=\frac{{\rm{\nu }}}{\varepsilon {^{\prime} }^{\mathrm{4/3}}}\frac{{\eta }^{2}+{\mathrm{(1}-\eta )}^{2}}{{\eta }^{\mathrm{1/3}}{\mathrm{(1}-\eta )}^{\mathrm{1/3}}}{(\frac{H}{{H}_{{\rm{crit}}}})}^{\mathrm{2/3}}\mathrm{.}$$

Here, ν and *η* are defined by the relations

6 $${\rm{\nu }}=-\frac{\alpha \,{\rm{Ai}}^{\prime} \mathrm{(0)}{({m}_{e}{c}^{2})}^{\mathrm{4/3}}}{\hslash },\,\eta =\frac{\varepsilon }{\varepsilon ^{\prime} },$$

with Ai′ being the derivative of the Airy function. The BE is a linear equation whose solution can be expressed in the general case with time-ordered exponentials^42^, and the dependence of these differential probabilities on the field strength *H* suggests the following assumption. If *H* is depending on time in two distinct systems, namely *H* = *H*_1_(*t*) in one system and *H* = *H*_2_(*t*) in the other, and *f*_*e*,*p*,*γ*_ are initially (*t* = 0) the same for both systems, the distribution functions will be also the same at time *t* for both systems if

7 $${\int }_{0}^{t}{H}_{1}^{\mathrm{2/3}}(t^{\prime} )dt^{\prime} ={\int }_{0}^{t}{H}_{2}^{\mathrm{2/3}}(t^{\prime} )dt^{\prime} \mathrm{.}$$

This *H*^2/3^-correspondence demonstrates that in the supercritical regime the spectra of particles with *χ* ≫ 1 can be modelled without the knowledge of the exact shape of the EM pulse. Also the *H*^2/3^-correspondence motivates the use of the global constant field approximation.

If ${t}_{{\rm{rad}}}\mathop{ < }\limits_{ \tilde {}}\tau$, the solution of Eqs (2) and (3) can be found in the framework of perturbation theory,

8 $$f(t)={f}^{\mathrm{(0)}}+{f}^{\mathrm{(1)}}+{f}^{\mathrm{(2)}}+\ldots \,,$$

where *f *^(*i*)^ ∝ (*t*/*t*_rad_)^*i*^ describes the *i*-th generation of the secondary particles. Substituting the initial energy distributions ${f}_{e}^{\mathrm{(0)}}=\delta (\varepsilon -{\varepsilon }_{0})$, ${f}_{p,\gamma }^{\mathrm{(0)}}=0$ into the right-hand-side of Eqs (2) and (3), one gets

9 $${f}_{e}^{\mathrm{(1)}}=t\cdot [{w}_{{\rm{rad}}}({\varepsilon }_{0}\to \varepsilon )-{W}_{{\rm{rad}}}\delta (\varepsilon -{\varepsilon }_{0})],$$

10 $${f}_{\gamma }^{\mathrm{(1)}}=t\cdot {w}_{{\rm{rad}}}({\varepsilon }_{0}\to {\varepsilon }_{0}-\varepsilon \mathrm{).}$$

It can be clearly seen from these formulas that from the experimental point of view it is highly desirable to have a very short EM pulse, *τ* ≪ *t*_rad_. In this case *f* ≈ *f *^(1)^, and the shapes of *f*_*e*,*γ*_(*ε*) directly reproduce the shape of *w*_rad_(*ε*_0_ → *ε*). Thus, measuring *f*_*e*,*γ*_(*ε*), one can find *w*_rad_(*ε*_0_ → *ε*), including the case $\alpha {\chi }^{\mathrm{2/3}}\mathop{ > }\limits_{ \tilde {}}1$.

In order to take into account the next terms of the perturbation theory, one considers the interval *ε*_0_/*χ*_0_ ≪ *ε* ≪ *ε*_0_, and obtains from Eqs (9) and (10) ${f}_{e}^{\mathrm{(1)}}(\varepsilon )\propto {\varepsilon }^{-\mathrm{1/3}}$, ${f}_{\gamma }^{\mathrm{(1)}}(\varepsilon )\propto {\varepsilon }^{-\mathrm{2/3}}$. By integrating over 1/*η* instead of *ε*′, it can be easily shown that any convolution from Eqs (2) and (3) for a power-law distribution function *f* ∝ *ε*^*s*^ yields again a power-law distribution with *f* ∝ *ε*^*s*−1/3^. The same holds for any multiplication by *W*_rad_ or *W*_pp_ in the BE, since *W*_rad_ ∼ *W*_pp_ ∝ *ε*^−1/3^. Thus, substituting ${f}_{e}^{\mathrm{(1)}}$ and ${f}_{\gamma }^{\mathrm{(1)}}$ into the right-hand-side of Eqs (2) and (3), one gets ${f}_{e,p,\gamma }^{\mathrm{(2)}}$, and, summarizing up to the second order of the perturbation theory on the interval where *χ* ≫ 1 and *ε* ≪ *ε*_0_, one finally has

11 $${f}_{e}\approx a{\varepsilon }^{-\mathrm{1/3}}+b{\varepsilon }^{-\mathrm{2/3}}+c{\varepsilon }^{-1},$$

12 $${f}_{p}\propto {\varepsilon }^{-1},\,{f}_{\gamma }\approx g{\varepsilon }^{-\mathrm{2/3}}-h{\varepsilon }^{-1},$$

where *a*, *b*, *c*, *g* and *h* are positive quantities.

For the proposed setup the EM pulse duration (*τ* ≈ 150 as) is about the radiation time (*t*_rad_ ≈ 200 as) and it is also about the characteristic time of pair production, so one expects a single generation of secondary particles from the peak of the pulse. However, the duration and the amplitude of the post- and prepulses [*τ* ∼ 3 fs and *E* ∼ 100, respectively, see Fig. 3(a,b)] are such that for the prepulse *t*_rad_ ∼ 1 fs and *τ*/*t*_rad_ ∼ 3, so one expects several generations of secondary particles from it.

### Approaching non-perturbative QED

Prior to entering the regime of fully non-perturbative QED, experiments will surely approach a perturbative supercritical regime. Such a regime is characterized by a large quantum parameter (*χ* ≫ 1), but perturbative QED calculations are still mostly correct so that the Nikishov–Ritus formulas are applicable. In the following, it is explained how one can identify the perturbative supercritical regime, and how one probably detect the break of perturbative QED, so enabling the determination of its range of validity. Therefore, a series of additional PIC simulations is performed in a one-dimensional geometry. Note that the attosecond pulse is now assumed to have a perfect temporal Gaussian profile, $a={a}_{0}\,{e}^{-{(x-ct)}^{2}/{\sigma }_{\tau }^{2}}$, instead of being generated in a self-consistent way. This kind of pulse is subsequently referred to as a clean Gaussian pulse. The additional simulations are also relevant in order to test the developed analytical model for a few generations of secondary particles, to test the *H*^2/3^-correspondence, and to figure out the role of the prepulse.

**Figure 5:**
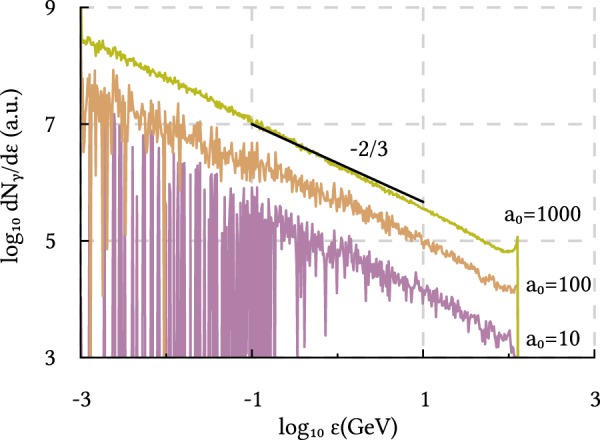
Spectrum of the photons after the interaction of a 125 GeV electron beam with a clean 150 as Gaussian EM pulse of different amplitudes *a*_0_ = 10, 100 and 1000. The black line shows the function *f* ∝ *ε*^−2/3^.

If *χ* ≫ 1, the Nikishov–Ritus formulas predict that the shape of the probabilities *w*_rad,pp_ remains the same for different field strengths, see Eqs (4) and (5). Simultaneously, the spectrum of the generated photon beam will reproduce the shape of *w*_rad_ if the EM pulse is short enough, as shown in the previous section. Thus, the shape of the photon spectrum remains the same when one increases the amplitude of an ultrashort EM pulse. This is in a good agreement with Fig. 5, where the photon spectra after the interaction of the electron beam with clean 150 as EM pulses of different strengths (*a*_0_ = 10, 100 and 1000) are given. The electron beam parameters remain unchanged (see the numerical modeling section for more information). Consequently, maximum values of approximately 12, 120 and 1200 are expected for *χ*. One can observe that all photon spectra obey the same power-law behavior that coincides with the behavior of the first generation of the photons predicted by Eq. (12). Thus, for ultrashort EM pulses, a change of the photon spectrum shape can indicate the entering in the regime of non-perturbative QED.

**Figure 6:**
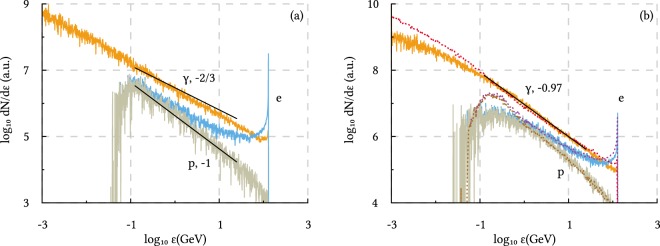
Spectrum of the photons (*γ*), electrons (e) and positrons (p) after the interaction of a 125 GeV electron beam with (**a**) a clean 150 as Gaussian EM pulse and (**b**) the EM pulse generated in the proposed setup (solid lines) and a clean 350 as Gaussian pulse (dashed lines). Black lines show the fits *f* ∝ *ε*^*s*^ on the interval from 125 MeV to 25 GeV, with *s* given in the labels.

To test the developed analytical model for a few generations of secondary particles, the interaction of the electron beam with clean attosecond EM pulses of various FWHM durations (*τ* = 50, 100, 150 and 350 as) and a fixed dimensionless field amplitude (*a*_0_ = 1450) is investigated in the following. Figure 6(a) shows the results for the collision with a clean Gaussian EM pulse that has a duration of 150 as. This duration is used since it matches the one of the generated main pulse [see Fig. 3(a)], and hence can be later used as a reference. Besides, Fig. 6(b) displays the results (solid lines) for the collision with the EM pulse that is generated in the laser-plasma interaction described in this manuscript. The additional black lines show the power-law fits *f* ∝ *ε*^*s*^ that are expected to describe the particle distribution function according to Eqs (11) and (12). The values of the power-law indexes are given in the labels. It can be seen in Fig. 6(a) that the power-law functions (11) and (12) are in reasonable agreement with the simulation results, indicating the regime of single generation of the secondary photons and positrons. However, note that the best power-law index for the photon distribution in Fig. 6(a) is approximately −0.77 which is close but not exactly equal to −2/3. This may indicate that the photon distribution function is already influenced by further generations of secondary photons. Therefore, simulations with shorter pulses are carried out. One obtains best-fitting power-law indexes of −0.73 and −0.67 for *τ* = 100 as and 50 as laser pulses, respectively.

If the EM pulse is not short enough, more generations of secondary particles should be taken into account. This can be seen, for instance, when returning to the photon spectrum that is obtained in the proposed setup [see solid lines in Fig. 6(b)]. A deviating power-law index indicates that the analytical solution (11) and (12) is inapplicable, and hence the pre- and postpulses of the attosecond pulse have to be included in the BE, e.g. by solving it numerically. However, the *H*^2/3^-correspondence allows one to reproduce the spectra of particles with *χ* ≫ 1 (*ε* ≫ 100 MeV) even in simple one-dimensional simulations, and so still enables a comparison of the experimental results and the predictions of perturbative QED. This can be achieved as follows. First, one has to retrieve the time integral over *H*^2/3^ along the electron trajectories for the attosecond pulse generated in the laser-plasma interaction. Then, one can find the duration of a clean Gaussian pulse that yields the same value of the time integral over *H*^2/3^. For the self-consistently generated attosecond pulse, this procedure leads to a duration of 350 as. The interaction of such a pulse with the electron beam results in particle spectra that coincide very good with the spectra obtained in the proposed setup, as can be seen from the dashed curves in Fig. 6(b). The comparison with the reference spectra in Fig. 6(a) finally gives insights into the impact of the prepulse on the distribution of high *χ* particles (*χ* ≫ 1, *ε* ≫ 100 MeV). Alternatively, the Gaussian pulse duration can be chosen to ensure this spectrum coincidence, for instance, when the time integral over *H*^2/3^ is not known for the generated attosecond pulse.

Therefore, if the duration of the EM pulse is such that a few generations of secondary particles arise, one can use the *H*^2/3^-correspondence to find the particle spectra in the framework of perturbative QED. Thus, variations in the emission and pair production probabilities caused by high-order radiative corrections will be detectable if they either disturb the *H*^2/3^-correspondence or the Ritus–Nikishov formulas.

## Conclusions

In conclusion, an experimental scheme has been proposed that might be promising for exploring a supercritical regime of radiation reaction or even a fully non-perturbative regime of QED. This scheme considers the generation of an ultraintense attosecond pulse via high harmonic generation at an over-dense plasma surface. In a second step, the EM attosecond pulse collides with a counterpropagating 100 GeV-class electron beam in a nearly head-on geometry. Numerical PIC simulations underline the feasibility of the proposal.

An analytical treatment reveals that if the attosecond pulse is short enough, the number of secondary particles will be small and the differential probability of the photon emission can be measured directly in the proposed scheme. In this regime the Ritus–Nikishov differential probabilities yield power-law spectra for the resulting photon and positron beams, *f*_*γ*_ ∼ *ε*^−2/3^ and *f*_*p*_ ∼ *ε*^−1^. Based on perturbative QED, the model further indicates that in the case of relatively long attosecond pulses the most part of the resulting particle spectra can be reproduced in simple one-dimensional simulations with clean Gaussian pulses of appropriate duration. If the Nikishov–Ritus formulas break, simple one-dimensional simulations will not be able to reproduce the experimental spectra any more, indicating the entering of the regime where high-order radiative corrections become important.

## Data Availability

The datasets generated during and/or analyzed during the current study are available from the corresponding author on reasonable request.
